# Platelets promote CRC by activating the C5a/C5aR1 axis via PSGL-1/JNK/STAT1 signaling in tumor-associated macrophages

**DOI:** 10.7150/thno.80555

**Published:** 2023-03-27

**Authors:** Xueqin Li, Xin Chen, Shengzhe Gong, Jie Zhao, Chen Yao, Hanyong Zhu, Rui Xiao, Yongqin Qin, Rongqing Li, Na Sun, Xiangyang Li, Fuxing Dong, Tingting Zhao, Yuchen Pan, Jing Yang

**Affiliations:** 1 Jiangsu International Laboratory of Immunity and Metabolism, Jiangsu Province Key Laboratory of Immunity and Metabolism, The Department of Pathogenic Biology and Immunology, Xuzhou Medical University, Xuzhou, Jiangsu, China; 2 Department of Clinical Laboratory, Affiliated Jinhua Hospital, Zhejiang University School of Medicine, Jinhua, China; 3 National Experimental Demonstration Center for Basic Medicine Education, Xuzhou Medical University, Xuzhou, Jiangsu, China; 4 Public Experimental Research Center, Xuzhou Medical University, Xuzhou, Jiangsu, China; 5 Chongqing International Institute for Immunology, Chongqing, China

**Keywords:** Platelets, TAMs, CRC, PSGL-1/JNK/STAT1, C5a/C5aR1.

## Abstract

**Rationale:** Platelets can influence the progression and prognosis of colorectal cancer (CRC) through multiple mechanisms, including crosstalk with tumor-associated macrophages (TAMs). However, the mechanisms underlying the crosstalk between platelets and TAMs remain unclear. The present study aimed to investigate the role of intratumoral platelets in regulating the function of TAMs and to identify the underlying mechanisms.

**Methods:** The interaction of platelets with macrophages was assessed in the presence or absence of the indicated compounds* in vivo*. An azoxymethane/dextran sodium sulfate (AOM/DSS)-induced CRC mouse model was used to investigate the role of platelets in controlling CRC development. Multiplexed immunofluorescence staining, fluorescence-activated cell sorting (FACS), and RNA sequence analysis were used to examine the changes in TAMs. TAMs and bone marrow-derived macrophages (BMDMs) were treated with the indicated compounds or siRNA against specific targets, and the expression levels of signal transducer and activator of transcription 1 (STAT1), c-Jun N-terminal kinase (JNK), and P-selectin glycoprotein ligand-1 (PSGL-1) were measured by Western blotting. The mRNA expression levels of complement 5 (*C5*), complement 5a receptor 1 (*C5ar1*), Arginase 1 (*Arg1*) and *Il10* were measured by real-time RT-PCR, and the complement 5a (C5a) concentration was measured by ELISA. The dual-luciferase reporter assay and ChIP assay were performed to examine the potential regulatory mechanisms of platelet induction of C5 transcription in TAMs.

**Results:** In our study, we found that an increase in platelets exacerbated CRC development, while inhibiting platelet adhesion attenuated tumor growth. Platelets signal TAMs through P-selectin (CD62P) binding to PSGL-1 expressed on TAMs and activating the JNK/STAT1 pathway to induce the transcription of C5 and the release of C5a, shifting TAMs toward a protumor phenotype. Inhibiting the C5a/C5aR1 axis or PSGL-1 significantly reduced CRC growth.

**Conclusions:** An increase in intratumoral platelets promoted CRC growth and metastasis by CD62P binding to PSGL-1 expressed on TAMs, leading to JNK/STAT1 signaling activation, which promoted C5 transcription and activated the C5a/C5aR1 axis in TAMs. Our study examined the mechanism of the crosstalk between platelets and TAMs to exacerbate CRC development and proposed a potential therapeutic strategy for CRC patients.

## Introduction

Colorectal cancer (CRC), which has a high morbidity rate, has become a global medical issue 1-3. Platelets play important roles in hemostasis and thrombosis, and thrombocytosis induced by cytokines could promote CRC progression, including cancer development and metastasis 4-6. Platelets extravasate and infiltrate the tumor and interact with CRC cells in a P-selectin (CD62P)-dependent manner, promoting epithelial-to-mesenchymal transition (EMT) and the adhesion of colorectal tumor cells to the endothelium, which triggers CRC progression 4-6. Therefore, increasing evidence has shown that platelet counts could be prognostic indicators for CRC patients 4, 7, 8, which suggests that inhibiting platelets might be a potential CRC treatment.

CD62P is expressed on platelets and was shown to activate complement via the alternative pathway in humans to establish an inflammatory environment 9. The complement system is a major component of the innate immune system and plays a crucial role in shaping the adaptive immune response and tumorigenesis. Under physiological conditions, the complement system merges at C3 to produce C5 invertase 10. Recent studies have shown that many complement components, such as C1q, C1s, C3 and properdin, complement activation and complement deposition are present in various tumors 11, 12, including liver metastases in patients with colorectal cancer 13. Most experimental models agree on the tumor-promoting effects of complement 3a (C3a) and complement 5a (C5a) 10. Deficiencies in complement C5 or complement 5a receptor 1 (C5aR1) but not C3 prevent CRC tumorigenesis 14 and metastasis 15. Additionally, C5 is highly expressed in the majority of liver metastases in CRC and promotes proliferation, migration, and invasion in CRC cell lines 16. Importantly, intratumoral C5a levels could be key determinants of the immunosuppressive tumor microenvironment 10. However, the mechanisms underlying platelet activation of C5a/C5aR1 in CRC remain to be further investigated.

It has been shown that macrophages can secrete C5a under stress conditions 17, leading to myeloid-derived suppressor cells (MDSCs) infiltration into the tumor microenvironment (TME) via its receptor C5aR1, which results in TME suppression 2. Inhibiting the C5a/C5aR1 axis re-educates tumor-associated macrophages (TAMs) to switch from a protumoral to antitumoral phenotype 16, abrogating tumor growth 2, 18.

Platelets orchestrate the monocyte/macrophage response to pathological conditions 19, 20. Compared with those of circulating monocytes from wild-type mice, the levels of inflammatory factors are higher in circulating monocytes from *Tpor^-/-^* mice in which platelets are deficient 21. Moreover, intra-alveolar platelets are indispensable for alveolar macrophage transcriptional reprogramming and polarization, leading to neutrophil clearance and the effective resolution of pulmonary inflammation 20, 22. TAMs that mainly originate from circulating monocytes are one of the most abundant immune cells in the CRC microenvironment 23. In a prostate cancer model, platelet-derived growth factor (PDGF) activated and polarized TAMs to the M2 phenotype, which in turn induced the growth of prostate cancer cells 24.

P-selectin glycoprotein ligand-1 (PSGL-1), which is the ligand of CD62P, is a member of the adhesion molecule family and has been recognized as a regulator of immune responses by myeloid cells 25-27. O-Glycosylation of PSGL-1 and CD62P induces the phosphorylation of Akt/mTOR and IκBα/NFκB in monocytes and promotes monocyte adhesion 28, while PSGL-1 deficiency attenuates leukocyte infiltration 29. By binding to PSGL-1, platelet-derived CD62P could shift macrophages toward an anti-inflammatory and protumor phenotype 19, 20. PSGL-1 appears to bridge platelet and macrophage functions. However, whether and how platelets educate TAMs by inducing TAM activation of C5a/C5aR1 axis in CRC remains unclear.

Therefore, the present study aimed to investigate the role of intratumoral platelets in regulating the C5a/C5aR1 axis and the function of TAMs, and to identify the underlying mechanisms. We found that intratumoral platelets triggered C5 expression in TAMs by mediating the c-Jun N-terminal kinase (JNK) signal transducer and activator of transcription 1 (STAT1) signaling pathway in a PSGL-1-dependent manner. Platelets induced C5aR1 expression by TAMs, which reduced TAM phagocytosis and endowed TAMs with a protumoral phenotype.

## Methods

### Mice and ectopic tumor implantation

*Aurka^fl/fl^*;*Cd19^Cre/+^* was generated and identified as described previously 30. C57BL/6 (B6) (CD45.2) mice were obtained from Shanghai Model Organisms Center, Inc. (Shanghai, China). *Rag2^-/-^* mice were obtained from Jackson Laboratory (Ellsworth, Maine, US). According to the protocol approved by the Institutional Animal Care and Use Committee of Xuzhou Medical University (202105A153), the mice were strictly maintained and housed under specific pathogen-free (SPF) conditions. Six- to eight-week-old age- and sex-matched mice were used for the animal experiments.

The flanks of the mice were shaved, and MC-38 cells (1 × 10^6^) were subcutaneously injected. When palpable tumors formed, the tumor size was measured thrice a week using calipers as described previously 31. At the end of the experiment, the animals were sacrificed, and the tumor weights were measured after careful resection. The tumor tissues were collected for further analysis.

B-cell adoptive transfer experiments were performed as previously described 32. CD19^+^B220^+^ B cells were isolated from the spleens of wild-type mice. The cells were collected and washed twice with PBS. Recipient mice were administered 2 × 10^6^ B cells every 7 days by tail vein injection.

Platelet induction experiments were performed as follows: 6-week-old female wild-type mice were subcutaneously injected with 100 ng/g murine TPO or the same volume of 0.1% bovine BSA on Day -4, -3, -2, and Day 7. Peripheral blood was collected, and the number of platelets was counted.

Platelet transfusion experiments were performed as described 33. Platelets (2 × 10^8^) were transfused via the tail vein into wild-type mice every 3 days for a total of 6 times.

For macrophage depletion, 200 μg anti-colony stimulating factor 1 receptor (CSF1R) Ab or isotype control Ab in PBS was intraperitoneally (i.p.) injected every other day from -3 days as previously described 34.

For the clopidogrel experiment, 5 mg/kg clopidogrel or the same volume of PBS was administered by oral gavage every other day when palpable tumors formed.

For anti-C5aR1 monoclonal antibody treatment, the mice were divided into two groups and treated with either the same volume of isotype control Ab or 1 mg/kg anti-C5aR1 mAb by intravenous injection twice every 7 days.

For anti-PSGL-1 monoclonal antibody treatment, the mice were divided into two groups and treated with either the same volume of isotype control Ab or 200 μg anti-PSGL-1 mAb by intraperitoneal injection every 4 days for a total of 4 times.

Colorectal cancer associated with colitis was induced as described previously 31. Briefly, 12-week-old male mice were intraperitoneally injected with 10 mg/kg AOM (Sigma-Aldrich), followed by treatment with 2% DSS (MW 36,000-50,000 Da; MP Biomedicals) in drinking water. Seven days later, mice were treated with normal drinking water for 14 consecutive days. The DSS treatment was repeated for three cycles. At the end of the experiment, the intestinal and colon sections were removed, washed with PBS, and opened longitudinally for analysis.

### Detection of lung metastasis

For the CRC syngeneic metastasis model, 6-week-old *Aurka^fl/fl^* or *Aurka^fl/fl^;Cd19^Cre/+^* mice were injected with MC-38 cells (2 × 10^6^) via the tail vein. The survival of the mice was tested. In addition, mice were sacrificed for an India ink experiment on the 10^th^ day to observe lung metastasis. India ink (15% black ink, in PBS, LEAGENE, Beijing, China) was injected into the exposed trachea of sacrificed mice to visualize macroscopic MC-38 lung metastatic nodules. Then, the lungs were dissected and fixed in a destaining solution (3.7% formaldehyde, 1.7% acetic acid in 70% ethanol) overnight, followed by image acquisition. On the 10^th^ day, mice were sacrificed and lung tissues were removed. Lung metastasis were also examined by HE staining.

### Cell isolation and culture

Bone marrow-derived macrophages (BMDMs) were cultured in DMEM supplemented with 10% FBS and 20 ng/mL murine recombinant macrophage-colony stimulating factor (M-CSF, Cloud-Clone Crop, USA) for 4-7 days as previously described 35. RAW264.7 cells were purchased from FuHeng (FuHeng Cell Center, Shanghai, China). 293T cells and RAW264.7 cells were cultured in DMEM containing 10% FBS at 37°C in a 5% CO_2_ environment.

### Reagents

SP600125, the platelet inhibitor clopidogrel, and murine TPO were obtained from MCE (Shanghai, China). Murine CD62P was purchased from Sino Biological (Beijing, China). The isotype antibody (Ab), anti-C5aR1 Ab, and anti-PSGL-1 Ab were purchased from Biolegend (San Diego, CA, USA) and Bio X cell (Lebanon, NH, USA).

### siRNAs and transfection

siRNAs against STAT1#1 (5'-GCUGGCCCUGAUGGUCUUATT-3', 5'-UAAGACCAUCAGGGCCAGCTT-3'), STAT1#2 (5'-GCUGUUACUUUCCCAGAUATT-3', 5'-UAUCUGGGAAAGUAACAGCTT-3'), PSGL-1#1 (5'-GCCACACAGUGGAGUCUAATT-3',5'-UUAGACUCCACUGUGUGGCTT-3') and PSGL-1#2 (5'-GGCCAUCCGUGACUCACUUTT-3', 5'-AAGUGAGUCACGGAUGGCCTT-3') were obtained from Jima (Shanghai, China). BMDMs were transfected with 1.2 μg of the indicated siRNAs in the presence of jetPRIME transfection reagent (Polyplus, France) according to the manufacturer's protocol. Twenty-four hours later, the transfected cells were subjected to the indicated experiments.

### Lentivirus infection

Human STAT1 lentivirus was obtained from Geme (Shanghai, China). RAW264.7 cells were infected with the empty or STAT1 lentivirus in the presence of 10 μg/mL polybrene. The infected cells were selected with puromycin.

### Platelet counts and preparation

Platelets were prepared and counted with a Sysmex XP-100 Hematologic Analyzer (Sysmex Corporation) as previously described 36.

### Tissue digestion and preparation of single-cell suspensions

After the experiment, tumor tissues were removed, and the tissues were cut into 5 mm-thick strips and placed into a digestive solution containing 2 mg/mL collagenase IV (VICMED, Xuzhou, China) and 0.25 mg/mL hyaluronidase (MCE) in RPMI-1640 culture medium (KeyGEN Biotechnology Co, Nanjing, China) 37. The tissues were incubated at 37 °C with continuous shaking at 180 rpm for 60-120 min and a gentle vortex every 15 min. The resulting cell suspensions were strained through a 70 μm cell strainer, pelleted, washed twice in PBS and sorted by flow cytometry 38.

### Surface staining, flow cytometry, and cell sorting

To analyze cells in tumor tissues, after being strained through a 70 μm cell strainer, the cells were stained with 7-AAD (559925, BD Bioscience) to label dead cells and stained for 15 min with Percp-Cy5.5-anti-CD45 (IC: PerCP-Cy5.5-IgG2b, κ, 30-F11, BD Biosciences, CA, USA, 1: 100), PE-anti-CD8 (IC: PE-Rat IgG2a, κ, 553032, BD Biosciences, 1: 200), BV421-anti-Ly6G (IC: BV421-Rat IgG2a, κ, 1A8, Biolegend, 1: 200), PE-anti-F4/80 (IC: PE-Rat (WI) IgG2a, κ, 565410, BD Bioscience, 1: 200), APC-anti-CD86 (IC: APC-Rat IgG2a, κ, E-AB-F0994UE, Elabscience, 1:200), Alexa-Fluor647-anti-CD206 (IC: Alexa Fluor647-Rat IgG2a, κ, BD Bioscience, 1: 200), or APC-anti-C5aR1 (IC: APC -IgG2b, κ, cat 135807, BD bioscience, 1: 200). Then, 10 μL of beads (Thermo) were added to each sample and subjected to FACS analysis.

For BMDMs analysis, after being challenged with the indicated treatment, BMDMs were harvested, stained with APC-anti-C5aR1, APC-CD86, or Alexa-Fluor647-anti-CD206 and subjected to FACS.

The data were analyzed using FlowJo (FlowJo LLC).

### Phagocytosis assay

Sorted TAMs were plated and incubated with 1 mg/mL FITC-dextran (MCE, Shanghai, China) or RB-dextran (Ruixibio, Guangzhou, China) at 37 °C for 1 h. Then, the cells were collected and subjected to flow cytometry to analyze phagocytosis.

### Dual luciferase activity assay

Luciferase assays were performed as previously described 39. Briefly, 2500 ng of empty firefly luciferase reporter vector or an equal amount of the human *C5* promoter-driven firefly luciferase reporter plasmid was transfected into cells with 4 µL/well jetPRIME transfection reagent (Polyplus, France). A pRL-TK plasmid (50 ng) was cotransfected as a control to determine transfection efficiency. Luciferase activity was measured for 10 sec in a luminometer. The *C5* promoter activity of each construct is expressed as firefly luciferase/Renilla luciferase activity.

### Chromatin immunoprecipitation (ChIP) assay

The binding site of STAT1 on the *C5* promoter was predicted by LASAGNA-Search 2.0 to search for transcription factor-binding sites (TFBSs) (https://biogrid-lasagna.engr.uconn.edu/lasagna_search). ChIP was performed using a commercially available kit (Beyotime Biotechnology) as previously described 40. Briefly, DNA-bound proteins were crosslinked using formaldehyde at a final concentration of 1% for 10 min at 37 °C, followed by immunoprecipitation using an anti-STAT1 antibody (#14994, CST). The DNA was extracted and subjected to PCR. The primer sets used to amplify the C5 promoter region between -1015 and -1000 bp were as follows: forward 5'-TGGCCTCCCTGTAAC-3' and reverse 5'-GTTACAGGGAGGCCA-3'. The PCR products were separated by electrophoresis in a 2% agarose gel and visualized by ethidium bromide staining.

### Correlation analysis using the GEPIA web tool

The online database Gene Expression Profiling Interactive Analysis (GEPIA2, http://gepia2.cancer-pku.cn/#index) was used to perform the correlation analysis of the indicated genes. GEPIA2 was also used to analyze PSGL-1 expression in CRC patients and to analyze PSGL-1 expression in different stages of CRC.

### PSGL-1 expression

PSGL-1 mRNA expression in different human cells was examined with BioGPS (http://biogps.org).

### Microarray analysis

Equal numbers of F4/80^+^ cells (5 × 10^5^ cells) were sorted from the tumor tissue of wild-type or TPO-treated mice by FACS. Total RNA was extracted using Trizol reagent (Invitrogen) and quantified by an ND-2000 (NanoDrop Technologies). RNA quality was examined by a 2100 Bioanalyzer (Agilent Technologies). A transcriptome library was prepared with the Clontech-SMART-Seq^TM^ v4 Ultra^TM^ Low Input RNA kit for sequencing using 10 ng of total RNA. RNA extraction and quantification, transcriptome library preparation, and sequencing were performed by Majorbio (Shanghai, China).

### Real-time reverse transcription-polymerase chain reaction (RT-PCR)

Total RNA of TAMs was isolated using Cell-to-CT 1-step Power SYBR Green kit (Invitrogen) according to the kit manual. Total RNA of macrophages was extracted by Trizol (Invitrogen), cDNA was synthesized and subjected to real-time RT-PCR using SYBR Green I Master Mix (Roche Diagnostics Gmbh) on a Light Cycler 480 system (Roche Group) as previously described 30. The real-time RT-PCR results have been normalized to *actb*. The primers used for real-time RT-PCR are shown in Table 1.

### Immunoblotting

Immunoblot assays were performed as previously described 41. Briefly, cell lysates were extracted and separated on an 8% SDS-PAGE gel. After semidry transfer, the membranes were sequentially probed with the indicated antibodies. Anti-p-JNK (#4668S, 1:2000), anti-JNK (#9252S, 1:2000), anti-p-STAT1 (#9167, 1:2000), anti-STAT1 (#14994, 1:2000), anti-p-STAT3 (#9145, 1:2000), anti-STAT3 (#12640, 1:2000), anti-p-p38 (#4s11, 1:2000), anti-p38 (#8690, 1:2000), anti-p-ERK (#4370s, 1:2000), and anti-ERK (#4695s, 1:2000) were purchased from Cell Signaling Technology (MA, USA). Anti-ITGB1 (#A19072, 1:1000), anti-ITGB5 (#A2497, 1:1000), anti-ITGB4 (#A4596, 1:1000), and anti-ITGA5 (#A19069, 1:1000) were purchased from ABclonal. Anti-PSGL-1 (#23605-1-AP, 1:1000) and anti-β-actin (# 66009-1-Ig, 1:2000) were obtained from Proteintech.

### Multiplexed immunofluorescence staining and quantification

A formalin-fixed, paraffin-embedded CRC tissue microarray was purchased from Shanghai Outdo. After deparaffinization, endogenous peroxidase blockade, and antigen retrieval, the slide was incubated with primary Abs using a PANO 7-plex IHC kit (PANO, Beijing, China) according to the instructions. Anti-C5/C5a was purchased from ABclonal (#A8104, 1:50). Anti-CD68 (#25747-1-AP, 1:200), anti-CD41 (#24552-1-AP, 1:100), and anti-PSGL-1 (#23605-1-AP, 1:100) were purchased from Proteintech. Images were acquired and analyzed using TissueFAXs and StrataQuest tissue analysis software (TissueGnostics, Beijing, China) as described previously 30.

Tissues were fixed in 10% formalin and paraffin-embedded by Servicebio (Nanjing, China). The sections were stained with hematoxylin-eosin (Beyotime Biotechnology, Nantong, Jiangsu, China).

The sections (5 μm) were cut and stained with anti-CD41 (#24552-1-AP, Proteintech, 1:100), anti-F4/80 (#D2S9R, CST, 1:200), anti-CD86 (#E5W6H, CST, 1:200), and anti-T-cell immunoglobulin mucin protein 4 (TIM4, #12008-1-AP, Proteintech, 1:200) using a PANO 7-plex IHC kit 30. Images were obtained under a Leica STELLARIS 5 confocal microscope.

### Enzyme-linked immunosorbent assay (ELISA)

Serum concentrations of C5a were examined using ELISA kits (MM-0401M1, Jiangsu Meimian Industrial Co., Ltd, Yancheng, China) according to the manufacturer's instructions.

### Statistical analysis

The azoxymethane/dextran sodium sulfate (AOM/DSS) model and the experiments using anti-CSF1R or anti-PSGL-1 Abs were performed once. The other mouse experiments except the pilot experiment were repeated twice, and the data were combined. Statistical analysis of differences was performed using unpaired Student's t test or one-way ANOVA followed by multiple comparisons for more than two groups by Prism statistical analysis software (GraphPad Software, San Diego, CA). The association between platelet expression and patient clinicopathological parameters was evaluated by chi-square (χ2) analysis by SPSS software (IBM). The data are presented as the mean ± SEM. Significance is indicated as follows: ** *P* < 0.01, * *P* < 0.05, or n.s. for not significant.

## Results

### An increase in platelets promotes tumor growth and metastasis

**Figure 1:**
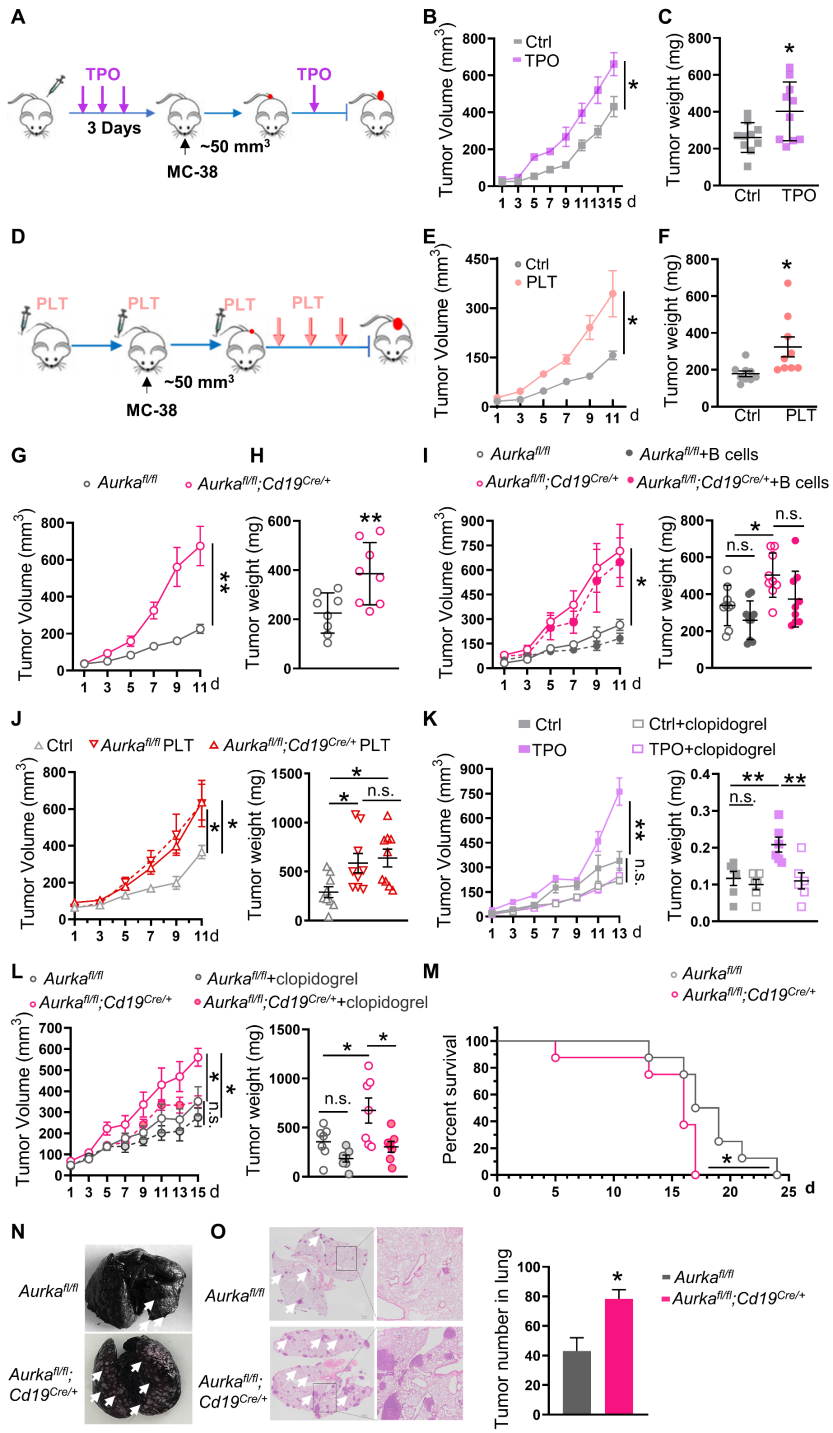
** An increase in platelets can promote the development of tumors. (A)** *In vivo* model of high platelet counts induced by four subcutaneous (s.c.) TPO injections. **(B)** Tumor growth was monitored (n = 10). **(C)** Tumor weights were examined (n = 10). **(D)** The increased platelet model established by six platelet transfusions. **(E)** Tumor growth in mice with or without platelet transfusion was monitored (n = 9). **(F)** The tumor weights were examined (n = 9). **(G)** Tumor growth in syngeneic CRC models established in *Aurka^fl/fl^* and *Aurka^fl/fl^;Cd19^cre/+^* mice was monitored (n = 8).** (H)** Tumor weights were examined (n = 8). **(I)** *Aurka^fl/fl^* and *Aurka^fl/fl^;Cd19^cre/+^* mice were transfused with the control or B cells. Tumor volumes and tumor weights were examined (n = 9). **(J)** Tumor growth in* Rag2^-/-^* mice transfused with or without platelets was monitored. Tumor weights were examined (n = 9).** (K-L)** Tumor-bearing mice were treated with the control or clopidogrel. The tumor growth and tumor weights were examined (K: n = 6, L: n = 7). **(M)** Survival was monitored 24 days after MC-38 injection. Statistical analysis of survival was performed with a log-rank test (n = 8). **(N)** Representative images of India ink-stained lungs with arrowheads indicating metastatic nodules (n = 3/ time point). **(O)** Representative H&E images of lung sections from *Aurka^fl/fl^* and *Aurka^fl/fl^;Cd19^cre/+^* mice injected with MC-38 cells. Scale bar, 2 mm, (n = 3). **P < 0.01; *P < 0.05; n.s., not significant.

To clarify the roles of platelets in CRC growth, mice were subcutaneously injected with TPO to increase platelet counts and intratumoral platelets (**Figure S1A-B**) and then transplanted with MC-38 cells. We found that a higher number of platelets promoted increases in tumor volume and tumor weight (**Figure 1A-C, Figure S1C**). In addition, the number of platelets was positively associated with tumor volume (**Figure S1D**). Similar results were obtained in wild-type mice that were adoptively transferred platelets (**Figure 1D-F, Figure S1E**). In addition, in the AOM/DSS-induced CRC model, tumor incidence was 100% in TPO-treated mice. However, only 3 of 6 control diluent-treated mice developed tumors (**Figure S1F**). In comparison with those of control diluent-treated mice, tumor number and size were increased in TPO-treated mice (**Figure S1G-I**). These results revealed that platelets could be one of the key mediators of tumor growth.

In our previous study, we established *Aurka^fl/fl^;Cd19^Cre/+^* mice, in which the development of B cells was impaired and the total number of platelets was increased without affecting platelet function 30, and we used *Aurka^fl/fl^* mice as matched controls. In the present study, the mice were transplanted with MC-38 cells. The increase in tumor growth was accelerated and there was an increase in the number of intratumoral platelets in *Aurka^fl/fl^;Cd19^cre/+^* mice compared with *Aurka^fl/fl^* mice (**Figure 1G-H, Figure S1J**), and these effects could not be rescued by a B-cell adoptive transfer experiment (**Figure 1I**). Furthermore, we adoptively transferred platelets isolated from *Aurka^fl/fl^* mice or *Aurka^fl/fl^;Cd19^cre/+^* mice. We found that tumor volumes and weights were increased in *Rag2^-/-^* mice that were administered *Aurka^fl/fl^* platelets and in mice that were administered *Aurka^fl/fl^;Cd19^cre/+^* platelets (**Figure 1J**). Conversely, the platelet adhesion inhibitor clopidogrel reduced tumor volumes and weights in wild-type mice treated with TPO (**Figure 1K**). Similar results were observed in *Aurka^fl/fl^;Cd19^cre/+^* mice (**Figure 1L**). Our data confirmed that an increase in platelets promoted CRC growth.

To study the role of platelets in CRC metastasis, we established an MC-38 syngeneic metastasis model. We found that all *Aurka^fl/fl^;Cd19^cre/+^* mice died within 17 days of being challenged with MC-38 cells, while 25% of *Aurka^fl/fl^* mice survived more than 20 days (**Figure 1M**). Visible metastatic lung nodules were observed and pulmonary metastasis increased within 10 days in *Aurka^fl/fl^;Cd19^cre/+^* mice (**Figure 1N-O**). Collectively, our results demonstrated that an increase in platelets mediated CRC growth and metastasis.

### An increase in platelets requires TAMs to drive tumor development

**Figure 2:**
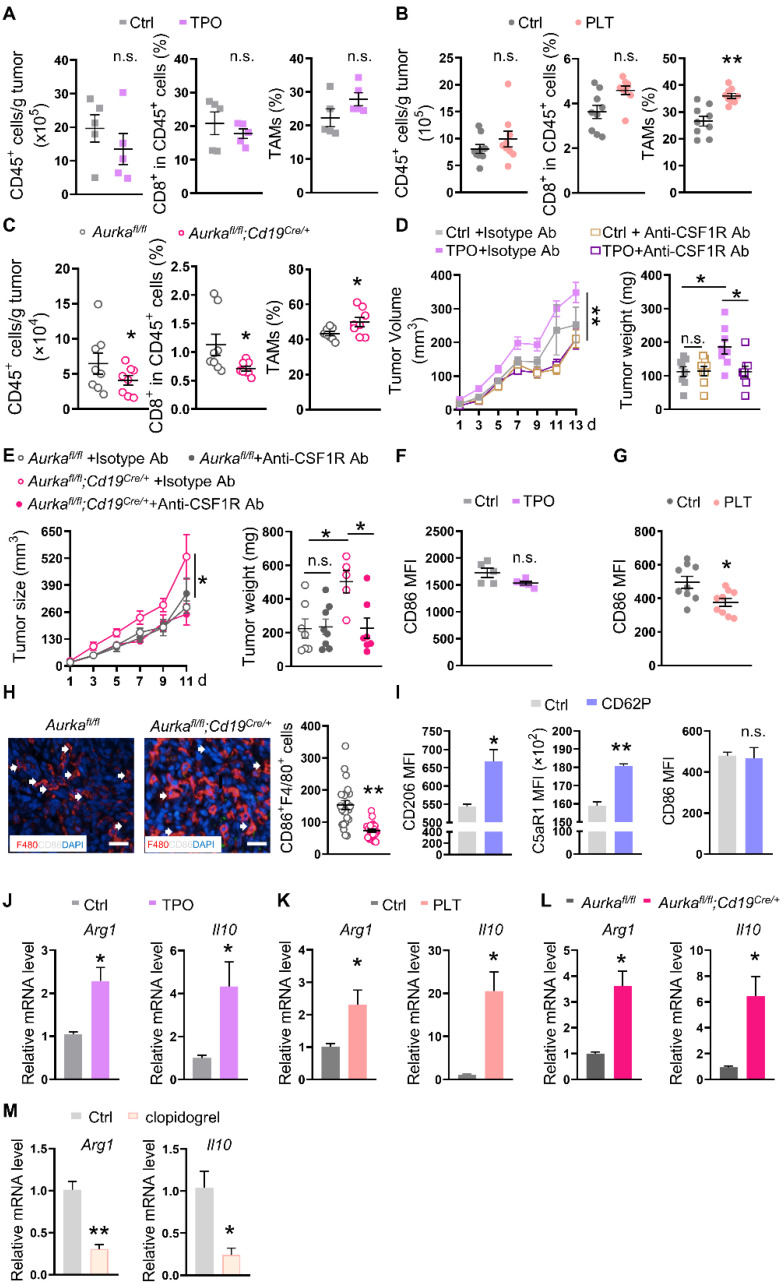
** Increased platelets rely on TAMs to drive tumor development. (A-C)** At the end of the experiment, the tumors were obtained, and single cells were prepared. The population of intratumoral CD45^+^ cells (left panel), the percentage of CD8^+^ T cells among CD45^+^ cells (middle panel), and TAMs among CD45^+^ cells (right panel) were investigated by FACS (A: n = 5 (another 5 tumor samples for TAM sequencing and C5aR1 analysis); B: n = 9; C: n = 8). **(D-E)** Tumor growth and tumor weights were examined (D: n = 8; E: *Aurka^fl/fl^* n = 7 (ctrl) and 8 (anti-CSF-1R), respectively, *Aurka^fl/fl^;Cd19^cre/+^* n = 5 (ctrl) and 7 (anti-CSF-1R), respectively). **(F-G)** The expression of CD86 on TAMs was analyzed by FACS (F: n = 5; G: n = 9). **(H)** Immunofluorescence analysis of F4/80 (red), CD86 (white), and DAPI (blue) in tumor sections from *Aurka^fl/fl^* and *Aurka^fl/fl^;Cd19^cre/+^* mice. The CD86^+^ F4/80^+^ cells were counted (n = 3 mice/group, 8 slides/mouse). **(I)** BMDMs were treated with or without CD62P for 6 h, after which, CD206, C5aR1 and CD86 were examined by FACS (n = 3). The data shown are representative of one of two independent experiments. **(J-M)** The mRNA expression levels of *Arg1* and* Il10* in TAMs isolated from mice bearing tumors were examined by real-time RT-PCR (n = 3). The data shown are representative of a single experiment. **P < 0.01; *P < 0.05; n.s., not significant.

Activated platelets not only influence tumor cells but also affect immune cells, including TAMs, by secreting multiple factors in the TME 42. To assess the roles of platelets in communicating with infiltrated immune cells, we examined the infiltrated immune cells and found that in comparison with those in tumors from control diluent-treated mice, the total number of CD45^+^ cells and the percentage of CD8^+^ T cells in CD45^+^ cells were almost identical in tumors harvested from mice that were treated with TPO (**Figure 2A**). Similarly, the total number of CD45^+^ cells and the percentage of CD8^+^ T cells in CD45^+^ cells did not change in the tumors from mice that were transfused with platelets (**Figure 2B**). However, CD45^+^ cell numbers and the percentage of CD8^+^ T cells were reduced in tumors from *Aurka^fl/fl^;Cd19^cre/+^* mice (**Figure 2C**). Compared with that in *Aurka^fl/fl^* tumors, the population of Ly6G^+^ neutrophils was almost identical in tumors from *Aurka^fl/fl^;Cd19^cre/+^* mice (**Figure S2A**). Unexpectedly, the percentage of TAMs tended to be increased in the tumors from the wild-type mice treated with TPO (**Figure 2A**). The percentage of TAMs was increased in the tumors from platelet-transfused mice (**Figure 2B**) and *Aurka^fl/fl^;Cd19^cre/+^* mice (**Figure 2C**).

To confirm whether elevated platelet-mediated tumor development depended on TAMs, we deleted TAMs *in vivo* using an anti-CSF1R antibody. We found that anti-CSF1R Ab treatment reduced tumor growth and tumor weight in TPO-treated mice but not in mice in the control group (**Figure 2D**). Similarly, anti-CSF1R treatment reduced tumor growth and tumor weight in *Aurka^fl/fl^;Cd19^cre/+^* mice but not in *Aurka^fl/fl^* mice (**Figure 2E**). These data revealed that an increase in platelets could promote tumor development in a TAM-dependent manner.

TAMs, especially M2-type TAMs, produce a large number of anti-inflammatory factors, including IL-10, which leads to an immunosuppressive microenvironment and orchestrates tumor growth 43. To determine whether platelets educated TAMs in the TME to promote tumor development, we sorted TAMs and examined key phenotypic markers. We found that the expression of CD86 tended to be reduced in TAMs isolated from the tumors of TPO-treated mice (**Figure 2F**). In addition, the CD86 expression was decreased in TAMs isolated from the tumors of platelet-transfused mice (**Figure 2G**). The number of CD86-positive TAMs was reduced in tumors from *Aurka^fl/fl^;Cd19^cre/+^* mice (**Figure 2H**). Consistently, the expression of CD206 and C5aR1 but not CD86 on BMDMs was elevated after the exposure of BMDMs to CD62P for 6 h (**Figure 2I**). In addition, we found that the number of TIM4-positive TAMs involved in mediating macrophage phagocytosis 44 was reduced in tumors from TPO-treated mice and platelet-transfused mice (**Figure S2B-C**). The phagocytic abilities of TAMs from *Aurka^fl/fl^;Cd19^cre/+^* mice were also decreased (**Figure S2D**). Real-time RT‒PCR revealed that the mRNA levels of Arginase 1 (*Arg1*) and *Il10* were increased in sorted TAMs from all three mouse models (**Figure 2J-L**). Conversely, clopidogrel decreased the mRNA levels of* Arg1* and *Il10* in sorted TAMs (**Figure 2M**). Collectively, these data indicate that an increase in platelets could drive tumor growth by shifting TAMs toward M2 polarization and impairing the phagocytosis of TAMs.

### The C5a/C5aR1 axis is critical for changes in TAMs induced by an increase in platelets

Under stress conditions, macrophages secrete C5a 18. Furthermore, inhibiting the C5a/C5aR1 axis could alter TAM polarization 16. We hypothesized that platelets could educate TAMs by activating the C5/C5aR1 axis.

**Figure 3:**
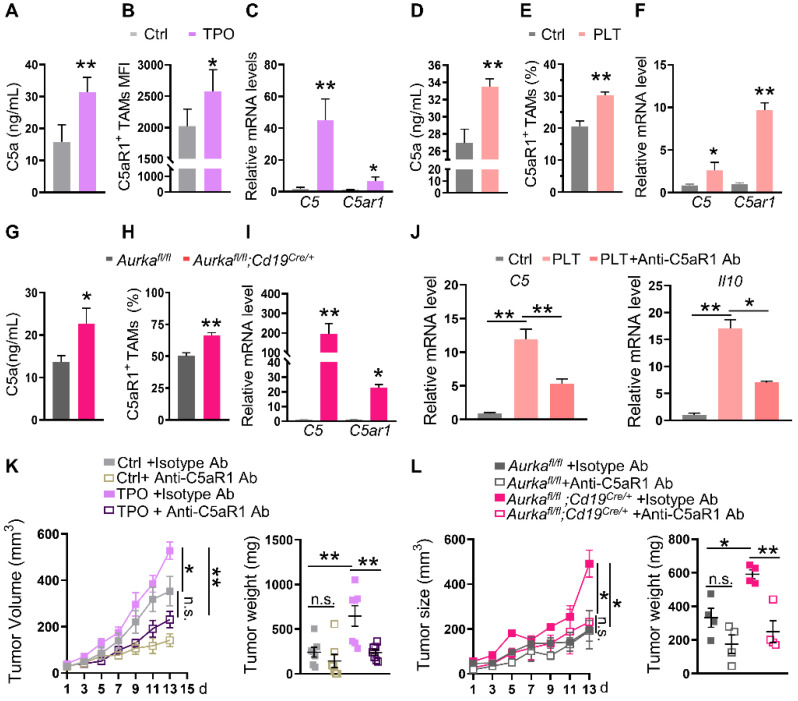
** Elevated platelet-regulated TAM education by the C5a/C5aR1 axis. (A)** The serum concentration of C5a was measured by ELISA (n = 5). The data shown are representative of one of two independent experiments.** (B)** The level of intratumoral C5aR1^+^ TAMs was examined by FACS (n = 5). **(C)** Real-time RT‒PCR was used to quantify the levels of *C5* and *C5ar1* mRNA (n = 3). (B-C) The data shown are representative of a single experiment. **(D)** The serum concentration of C5a was measured by ELISA (n = 5). The data shown are representative of one of two independent experiments. **(E)** The level of intratumoral C5aR1^+^ TAMs was examined by FACS (n = 9). **(F)** Real-time RT‒PCR was used to quantify the levels of *C5* and *C5ar1* mRNA (n = 3). (E-F) The data shown are representative of a single experiment. **(G)** The serum concentration of C5a was measured by ELISA (n = 7). The data shown are representative of a combination of two independent experiments.** (H)** The level of intratumoral C5aR1^+^ TAMs was examined by FACS (n = 7). **(I)** Real-time RT‒PCR was used to quantify the levels of *C5* and *C5ar1* mRNA (n = 3). (H-I) The data shown are representative of a single experiment. **(J)** Real-time RT-PCR was used to quantify the levels of *C5* and *Il10* mRNA after C5aR1 inhibition *in vitro*. The data shown are representative of one of two independent experiments. **(K-L)** Tumor volumes and tumor weights were examined (K: n = 7; L: n = 4). The data shown are representative of a single experiment. **P < 0.01; *P < 0.05; n.s., not significant.

To test this hypothesis, we examined C5a concentrations and the expression of C5aR1 in TAMs. We found that serum concentrations of C5a but not liver concentrations of C5a were increased (**Figure 3A, Figure S3A**). Of note, we found that the expression of C5aR1 on TAMs was elevated, which positively correlated with the number of platelets in TPO-treated mice (**Figure 3B, Figure S3B**). Moreover, the mRNA levels of *C5* and *C5ar1* were increased in TAMs (**Figure 3C**). Similar to the observations in TPO-treated mice, serum C5a concentrations but not liver C5a concentrations, C5aR1^+^ TAMs and the mRNA levels of *C5* and* C5ar1* were elevated in the platelet-transfused mouse model and in *Aurka^fl/fl^;Cd19^cre/+^* mice (**Figure 3D-I, Figure S3C-D).** Blocking C5aR1 inhibited the mRNA expression of *C5* and *Il10* mediated by platelets *in vitro* (**Figure 3J**). Inhibiting C5aR1 also attenuated MC-38 cell growth in wild-type mice treated with TPO and *Aurka^fl/fl^;Cd19^cre/+^* mice (**Figure 3K-L**). These results indicate that an increase in intratumoral platelets can promote tumor growth by switching the TAM phenotype to M2 via the activation of C5a/C5aR1 signaling.

### An increase in platelets requires PSGL-1 to promote C5 transcription

**Figure 4:**
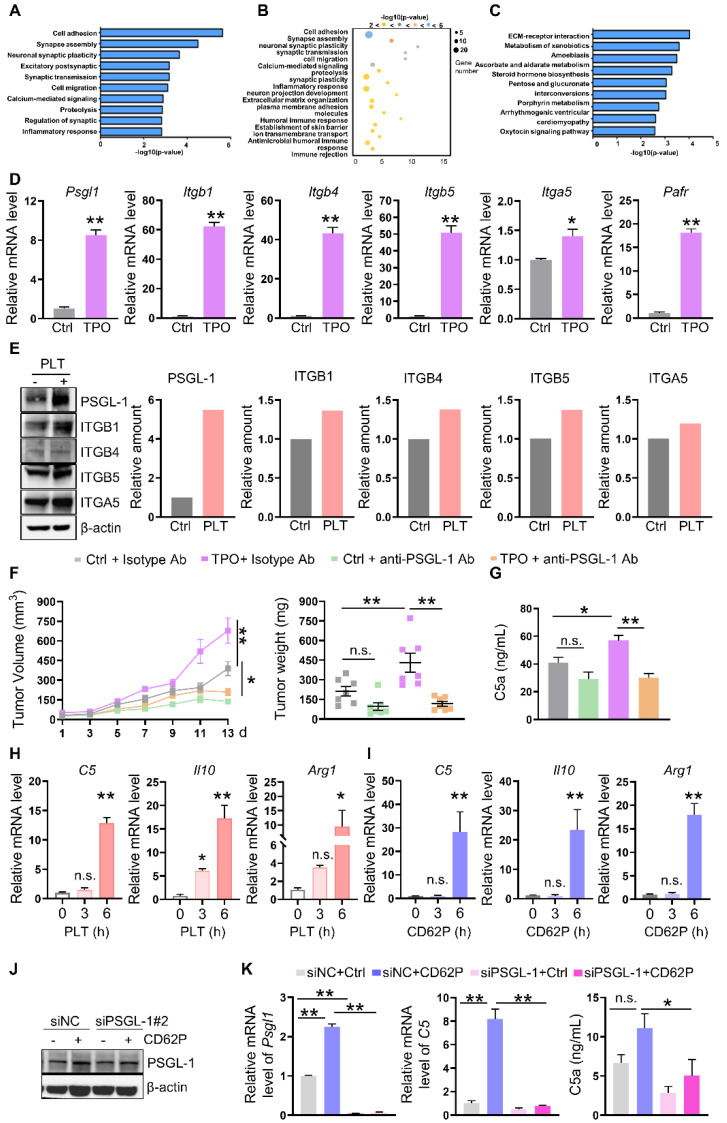
**Increased platelet-mediated promotion of C5 transcription requires PSGL-1. (A-B)** GO-BP analysis (cell adhesion) of the genes in TAMs isolated from mice treated with or without TPO. **(C)** KEGG analysis (cell adhesion) of the genes in TAMs isolated from mice treated with or without TPO. **(D)** Real-time RT-PCR was used to quantify the levels of *Psgl1*, *Itgb1*, *Itgb4*, *Itgb5*, *Itga5,* and *Ptafr* mRNA (n = 3). The data shown are representative of a single experiment. **(E)** BMDMs were treated with or without platelets for 6 h. Then, cell lysates were harvested and subjected to immunoblotting. **(F)** Tumor volumes and tumor weights were examined (n = 7). **(G)** The serum concentration of C5a was measured by ELISA (n = 7). (F-G) The data shown are representative of a single experiment. **(H-I)** The relative mRNA levels of *C5*, *Il10*, and *Arg1* were examined in BMDMs treated without or with platelets (H) or in the absence/presence of CD62P (I) for the indicated times. The data shown are representative of one of two independent experiments. **(J-K)** BMDMs were transfected with a negative control siRNA or an siRNA against PSGL-1. After 24 h, cell lysates were harvested and subjected to immunoblotting to examine the indicated proteins **(K)** and real-time RT-PCR to quantify the *Psgl1* and *C5* mRNA levels. The cell culture medium was harvested, and C5a was examined. (J-K) The data shown are representative of one of three independent experiments. **P < 0.01; *P < 0.05; n.s., not significant.

To demonstrate the underlying mechanisms of increased platelet-induced C5 transcription in TAMs, we sorted TAMs and performed RNA sequence analysis. Gene ontology biological process (GO-BP) analysis and KEGG analysis showed that the gene profiles were enriched in cell adhesion, synapse assembly, and ECM-receptor interaction (**Figure 4A-C**). Interestingly, the expression of genes involved in mediating adhesion, such as* Itgb1, Itgb4, Itgb5, Itga5,* and *Psgl1,* the ligand of P-selectin expressed on platelets, was increased in TAMs isolated from TPO-treated tumors (**Figure 4D**). The mRNA levels of the gene encoding platelet-activating factor receptor (PAFR) were also increased (**Figure 4D**). BMDMs were treated with platelets, and the protein expression of ITGB1, ITGB4, ITGB5, ITGA5, and PSGL-1 was induced, among them, PSGL-1 was the most increased protein (**Figure 4E**). To further verify the role of PSGL-1 in platelet-induced tumor growth, we established MC-38-bearing mice in the presence of TPO or control diluent and treated these mice with an isotype control Ab or anti-PSGL-1 Ab. We found that anti-PSGL-1 treatment inhibited tumor growth in parallel with a decrease in C5a concentrations (**Figure 4F-G**), indicating that PSGL-1 may be the key regulator of TAM polarization.

To determine whether the increased intratumoral platelets interacted with PSGL-1 expressed on TAMs via its receptor CD62P, BMDMs were treated with platelets or recombinant CD62P. As expected, in the presence of platelets or CD62P, the mRNA levels of *C5, Arg1,* and *Il10* were increased in a time-dependent manner (**Figure 4H-I**). We next treated BMDMs with CD62P in the presence or absence of siRNA against PSGL-1 and found that in the presence of CD62P, knocking down PSGL-1 decreased *C5* mRNA expression (**Figure 4J-K, Figure S3E-F**). C5a production have reduced tendency (**Figure 4K, Figure S3E-F**). These observations revealed that CD62P on platelets could activate C5a/C5aR1 signaling by binding to PSGL-1 on TAMs, inducing TAMs to polarize to the M2 phenotype.

### Platelets drive C5 transcription via PSGL-1-regulated JNK/STAT1 signaling

**Figure 5:**
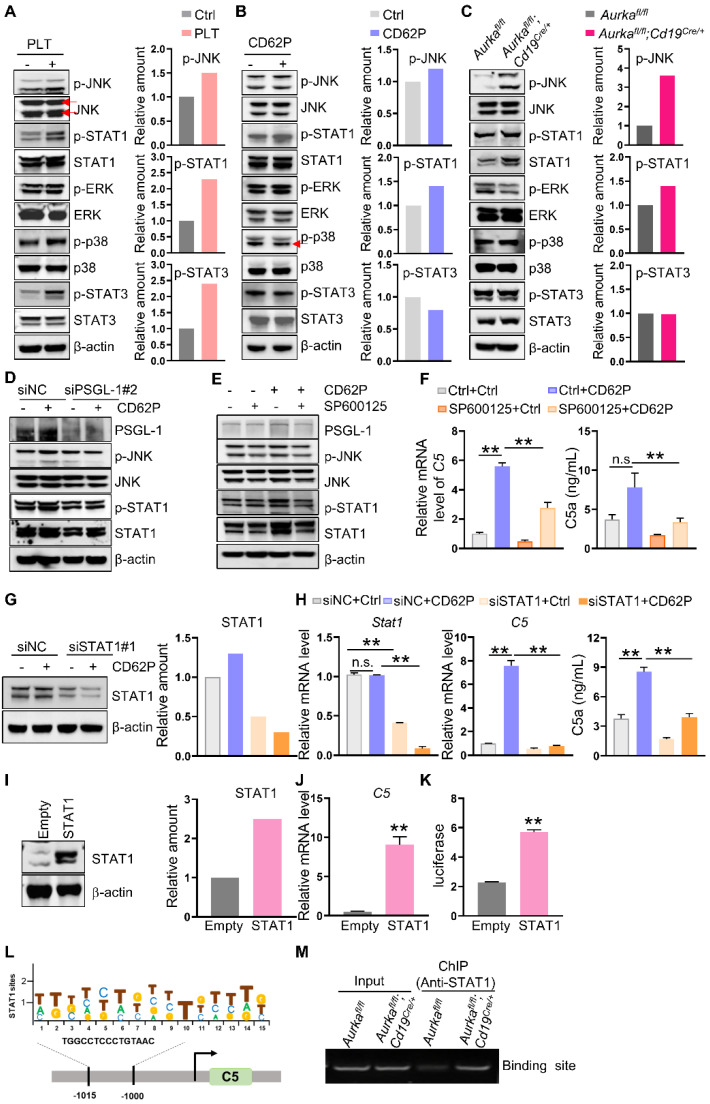
** Platelets regulate JNK/STAT1 signaling and drive C5 transcription in a PSGL-1 dependent manner. (A-C)** BMDMs were treated as indicated. After 6 h, total cell lysates were analyzed by immunoblotting. The levels of p-JNK, p-STAT1 and p-STAT3 were quantified by densitometry, normalized to actin, and plotted (right panel). **(D)** BMDMs were transfected with a negative control siRNA or an siRNA against PSGL-1 in the absence or presence of CD62P. After 24 h, cell lysates were harvested and subjected to immunoblotting. **(E)** BMDMs were treated with SP600125 and/or CD62P. After 18 h, cell lysates were harvested and subjected to immunoblotting. **(F)** BMDMs were treated with SP600125 and/or CD62P. After 18 h, RNA was extracted, and the levels of *C5* mRNA were examined by real-time RT-PCR. The cell culture medium was harvested, and C5a was examined (right panel). **(G)** BMDMs were transfected with a negative control siRNA or siSTAT1 in the presence or absence of CD62P. Twenty-four hours later, cell lysates were harvested and subjected to immunoblotting. The amount of STAT1 was quantified by densitometry, normalized to actin, and plotted (right panel). **(H)** BMDMs were treated with negative control siRNA or siSTAT1 in the presence or absence of CD62P. After 24 h, RNA was extracted and subjected to quantification of *Stat1* and *C5* mRNA levels by real-time RT-PCR. Cell culture medium was collected, and ELISA was used to examine the C5a concentration (right panel). **(I)** RAW264.7 cells were infected with empty or STAT1 lentivirus and selected with puromycin to establish stable cell lines. Cell lysates were extracted and subjected to immunoblotting. The levels of STAT1 were quantified by densitometry, normalized to actin, and plotted (right panel). **(J)** Real-time RT-PCR was used to quantify *C5* mRNA levels in RAW264.7-Empty and RAW264.7-STAT1 cells. **(K)** Luciferase assays were performed to verify the increase in *C5* transcriptional activity in 293T cells after the overexpression of STAT1. (A-K) The data are representative of one of two independent experiments.** (L)** STAT1 binds to the C5 gene promoter. Location and sequence of STAT1 response elements. **(M)** BMDMs were harvested and subjected to ChIP assays to examine STAT1 binding sites on the C5 promoter. bp, base pair. The data shown are representative of one of two independent experiments. **P < 0.01; n.s., not significant.

Next, we examined the signaling pathways, including JNK, ERK, and p38, in macrophages after platelet treatment. We found that in the presence of platelets, the expression of p-JNK, p-STAT1 and p-STAT3 but not p-ERK or p-p38 was increased in BMDMs (**Figure 5A**). On the other hand, the levels of p-JNK and p-STAT1 were also increased in BMDMs treated with CD62P and in BMDMs from *Aurka^fl/fl^;Cd19^cre/+^* mice (**Figure 5B-C**). siRNA-mediated downregulation of PSGL-1 reduced the expression of p-JNK and p-STAT1 mediated by CD62P (**Figure 5D, Figure S3E**), indicating that the JNK/STAT1 signaling pathway could be a key mediator of the effects of platelets on C5.

To confirm the roles of JNK and STAT1 in platelet-regulated C5 transcription, we first inhibited JNK activation using the specific inhibitor SP600125 and demonstrated that suppressing JNK activation reduced *C5* mRNA levels and p-STAT1 expression in CD62P-treated BMDMs (**Figure 5E-F**). SP600125 tended to diminish C5a production in CD62P-treated BMDMs (**Figure 5F**). Similarly, blocking JNK in *Aurka^fl/fl^* BMDMs or* Aurka^fl/fl^;Cd19^cre/+^* BMDMs inhibited the phosphorylation of JNK and STAT1 (**Figure S4A**). Silencing STAT1 attenuated CD62P-mediated C5 transcription and C5a concentration (**Figure 5G-H, Figure S4B-C**). On the other hand, overexpression of STAT1 increased *C5* mRNA levels (**Figure 5I-J**). We also found that STAT1 could directly bind to the *C5* promoter and enhance *C5* transcription (**Figure 5K-M**). Collectively, our data indicated that higher platelet counts could promote *C5* transcription via the JNK/STAT1 signaling pathway in macrophages.

### Intratumoral platelets are closely associated with C5a levels in CRC patients

**Figure 6:**
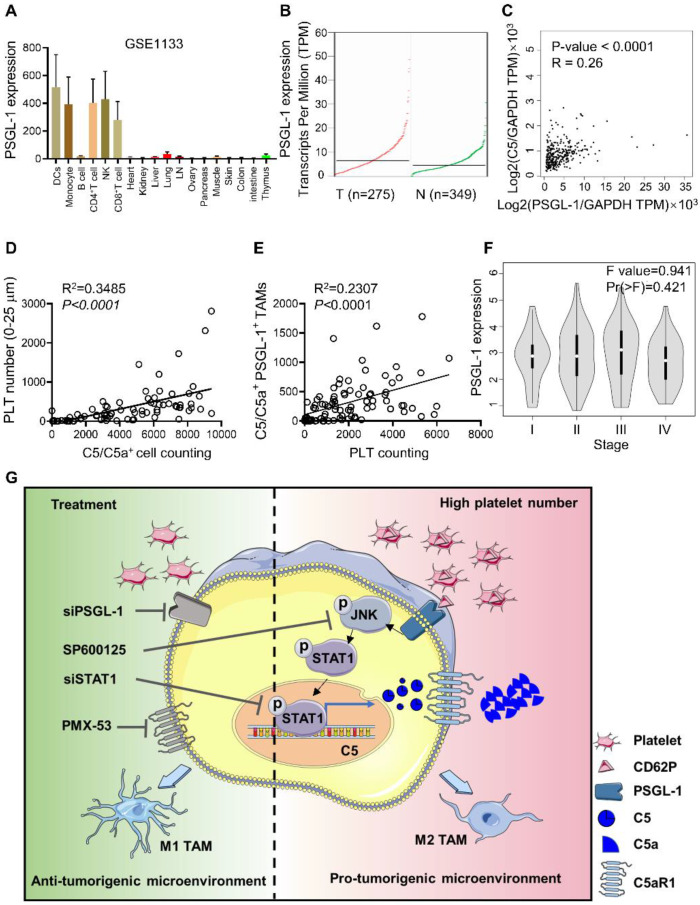
** Intratumoral platelet numbers are closely associated with C5a expression in TAMs. (A)** BioGPS was used to analyze *Psgl1* mRNA expression in different human cells. **(B)** GEPIA2 was used to analyze PSGL-1 expression in normal and CRC patients. **(C)** The correlation between PSGL-1 expression and C5 expression was analyzed by GEPIA2.** (D)** The correlation between C5/C5a-positive cells and the number of platelets surrounding C5/C5a^+^ cells within 25 μm was analyzed. **(E)** The correlation between platelet number and the number of C5/C5a^+^ PSGL-1^+^ CD68^+^ cells were analyzed. (D-E) The data shown are representative of a single experiment.** (F)** GEPIA2 was used to analyze PSGL-1 expression in different stages of CRC. **(G)** Schematic representation of platelet-driven C5 transcription via PSGL-1 regulated JNK/STAT1 signaling. In the CRC microenvironment, more platelets bound to PSGL-1 on TAMs via their CD62P and led to activation of the JNK/STAT1 signaling pathway, inducing C5 transcription and activation of the C5a/C5aR1 axis, which in turn shifted TAMs toward a protumoral phenotype and promoted tumor growth and metastasis.

PSGL-1 was mainly expressed in monocytes, T cells, and DCs but not in normal intestinal and colon tissues, and PSGL-1 expression was higher in CRC tissues than in normal tissues (**Figure 6A-B, Figure S5A**). We found that *Psgl1* expression was positively associated with *C5* expression, as determined by the GEPIA web tool (**Figure 6C**). To further verify the correlation between PSGL-1 and C5/C5a expression in CRC patients, we used a CRC tissue microarray and examined the colocalization of platelets and C5/C5a^+^ cells in CRC tissues (**Figure S5B**). We found that platelet numbers (surrounding C5/C5a^+^ cells within 25 μm) were positively associated with C5/C5a^+^ cell numbers (**Figure 6D, Table 2**). The number of platelets was also positively associated with the number of C5/C5a^+^PSGL-1^+^TAMs (**Figure 6E, Table 2**). Although PSGL-1 expression was not correlated with CRC stage (**Figure 6F**), It seemed that high number of intratumoral platelets was prone to be negatively associated with poor prognosis, although there was no positive correlation between the number of intratumoral platelets and tumor size (**Figure S5C-D**). Additionally, due to limited samples, no positive correlation was observed between the number of intratumoral C5/C5a^+^ cells and tumor size (**Figure S5E**), which should be further studied in a larger cohort. Taken together, our data indicated that in CRC patients, an increase in intratumoral platelets promoted TAM production of C5/C5a.

## Discussion

Chemokines in platelet α-granules have been generally accepted as effectors of inflammation and immunity 4, 45, 46. Blocking platelets with clopidogrel or ticagrelor reduced the growth and metastasis of CRC 47 and pancreatic cancer 48. On the other hand, intratumoral platelets promoted CRC metastasis by driving EMT *in vivo*, indicating that platelets could modulate tumor behavior 4. In this study, we found that an increase in platelets promoted tumor growth and metastasis in parallel with an increase in C5a production, which was critical for tumorigenesis (Figures 1-3).

Macrophages are one of the main sources of C5, and TAMs are one of the most abundant immune cells in the CRC microenvironment 49, suggesting that TAMs may be the most important source of C5 in the CRC microenvironment. C5aR deficiency upregulated the expression of the proinflammatory factors NOS2 and IL-1β in macrophages, and downregulated the anti-inflammatory factors Arg-1, IL-10 and TGF-β 15. On the other hand, by binding to its receptor C5aR1, C5a activation induces macrophage M2 polarization via the chemokine CCL2 50. Furthermore, C5a can suppress cytotoxic T-cell function by recruiting MDSCs to the tumor microenvironment. C5a also promoted hepatic metastasis of colon cancer through NF-κB signaling 51. Collectively, the C5a/C5aR axis plays an important role in regulating M2 macrophage polarization and the behavior of CRC cells.

In the present study, platelets upregulated *C5* mRNA and C5a levels in TAMs via the JNK/STAT1 signaling pathway. However, blocking STAT1 with siRNA or suppressing JNK with its inhibitor SP600125 did not completely decrease C5 expression in macrophages (Figure 5). Reports have shown that other receptors expressed by platelets, including GPVI, could participate in the interaction with macrophages 52, suggesting that other signaling pathways may regulate complement production in macrophages. C5 function could be regulated by ERK 53 and STAT3 signaling 54. However, the upstream regulation of C5 is unknown.

It has been reported that platelets interact with monocytes via CD11a/b/c/CD18 binding and upregulate proteins that indicate platelet activation 55. In the present study, we found that C5 was elevated in macrophages when they were directly or indirectly cocultured with live platelets, while C5 expression remained unchanged in macrophages that were treated with platelet lysates (data not shown). We hypothesized that there could be a feedback loop between platelets and macrophages. However, the mechanism of this feedback loop is still unclear.

Platelets can release multiple cytokines to mediate the functions of immune cells 56. Platelet-derived TGF-β contributes to the pathogenesis of pulmonary arterial hypertension 57, and TGF-β contributes to the malignant transformation of the tumor microenvironment 58. Furthermore, TGF-β is beneficial for establishing an immunosuppressive microenvironment 59, which promotes macrophage polarization to the M2 phenotype 60 and accelerates tumor development. Platelets can also deliver microRNAs to tumor cells 61 and nearby immune cells 62. Engineered platelets can be efficient and efficacious in suppressing tumor proliferation 63. Overall, the determination of key factors in platelets that mediate TAMs is important for tumor treatment.

In the present study, we found that an increase in platelets in the CRC microenvironment promoted tumor growth and metastasis by CD62P binding to PSGL-1 on TAMs, which shifted TAMs toward a protumor phenotype via the C5a/C5aR1 axis. Inhibiting PSGL-1 or C5aR1 inhibited CRC growth in mice. Our study provides new insight into platelet functions in modulating the tumor microenvironment. Targeting intratumoral platelets could be a therapeutic strategy for CRC patients.

## Conclusions

In summary, our work showed that platelets drive* C5* transcription in TAMs by activating the PSGL-1/JNK/STAT1 signaling pathway, contributing to M2 education by the C5a/C5aR1 axis.

## Supplementary Material

Supplementary figures.

## Figures and Tables

**Table 1:** Real-time RT-PCR primers.

Gene	Direction	Primer
*Arg1*	Forward	5'-GACCTGGCCTTTGTTGATGT-3'
	Reverse	5'-CAGCTCTTCATTGGCTTTCC-3'
*C5*	Forward	5'-GCTAGCCTTCACACCTCCAG-3'
	Reverse	5'-CAGGGTGAAGGTCACCAAGT-3'
*C5ar1*	Forward	5'-GATGCCACCGCCTGTATAGT-3'
	Reverse	5'-ACGGTCGGCACTAATGGTAG-3'
*II10*	Forward	5'-ACTGCTATGCTGCCTGCTCT-3'
	Reverse	5'-TCTAGGAGCATGTGGCTCTG-3'
*Itgb1*	Forward	5'-ggtgtcgtgtttgtgaatgc-3'
	Reverse	5'-tgacgctagacatggaccag-3'
*Itgb4*	Forward	5'-gaaggagttgcaggtgaagc-3'
	Reverse	5'-gctgagttggacttggaagc-3'
*Itga5*	Forward	5'-agcgcatctctcaccatctt-3'
	Reverse	5'-aggcattgaggcagaagcta-3'
*Itgb5*	Forward	5'-tgggtagacaccatcgtcaa-3'
	Reverse	5'-tgggcagttctgtgtagctg-3'
*Psgl1*	Forward	5'-AACTACTCCCCCACGGAGAT-3'
	Reverse	5'-ctgggctctgtcttcaggtc-3'
*Pafr*	Forward	5'-AGCAGAGTTGGGCTACCAGA-3'
	Reverse	5'-GACACAGTTGGTGCTGAGGA-3'
*Actb*	Forward	5'-GCTACAGCTTCACCACCACA-3'
	Reverse	5'-TCTCCAGGGAGGAAGAGGAT-3'

**Table 2:** Platelet count and clinicopathological characteristics in CRC patients.

Variables	platelet (n = 90)				
	Total	High (%)	Low (%)	N/A	*P*
All patients	90	45(100)	44(100)	1(100)	
Gender					0.913
Males	47	23	23	1	
Females	43	22	21	0	
Age (years)					0.545
< 60	30	16	13	1	
≥ 60	60	29	31	0	
Depth of infiltrations					0.314
T1+T2	12	4	7	1	
T3+T4	78	41	37	0	
Regional Lymph				0	0.943
N0 (negative)	62	31	30	1	
N1+N2 (positive)	28	14	14	0	
Distant metastasis					0.544
M0 (negative)	87	44	42	1	
M1+M2 (positive)	3	1	2	0	
Length of Tumor (cm)					0.730
< 5	37	19	17	1	
≥ 5	53	26	27	0	
C5a^+^ Cells					0.000
1-3000	33	3	29	1	
3001-6000	28	16	12	0	
> 6001	29	26	3	0	
CD68^+^ Cells					0.004
< 537	46	16	29	1	
≥ 537	44	29	15	0	
CD68^+^PSGL-1^+^ Cells					0.071
0-300	54	22	31	1	
301-500	18	10	8	0	
> 501	18	13	5	0	
CD68^+^ PSGL-1^+^ C5/C5a^+^ Cells					0.026
≤ 220	45	17	27	1	
> 221	45	28	17	0	

## Abbreviations

Arg1: Arginase-1
AOM: azoxymethane
BMDMs: Bone marrow-derived macrophages
C5aR1: Complement 5a receptor 1
C5a: complement 5a
CRC: Colorectal cancer
CSF1R: colony-stimulating factor 1 receptor
DSS: dextran sodium sulfate
EMT: epithelial-to-mesenchymal transition
FACS: Fluorescent-activated cell sorting
GO-BP: Gene ontology biological processes
IL-10: Interleukin -10
JNK: c-Jun N-terminal kinase
MDSCs: Myeloid-derived suppressor cells
M-CSF: murine recombinant macrophage-colony stimulating factor
PDGF: platelet-derived growth factor
PSGL-1: P-selectin glycoprotein ligand-1
STAT1: signal transducer and activator of transcription 1
TAMs: Tumor-associated macrophages
TGF-β: transforming growth factor β
TME: tumor microenvironment
TIM4: T-cell immunoglobulin mucin protein 4
