# Supplementary material for: Platelets promote CRC by activating the C5a/C5aR1 axis via PSGL-1/JNK/STAT1 signaling in tumor-associated macrophages
Source: Theranostics. 2023 Mar 27;13(6):2040–56. doi: 10.7150/thno.80555 (PMC10091882; doi:10.7150/thno.80555)
Supplement: Supplementary file 1 — Supplementary figures. [file thnov13p2040s1.pdf]

Figure S1

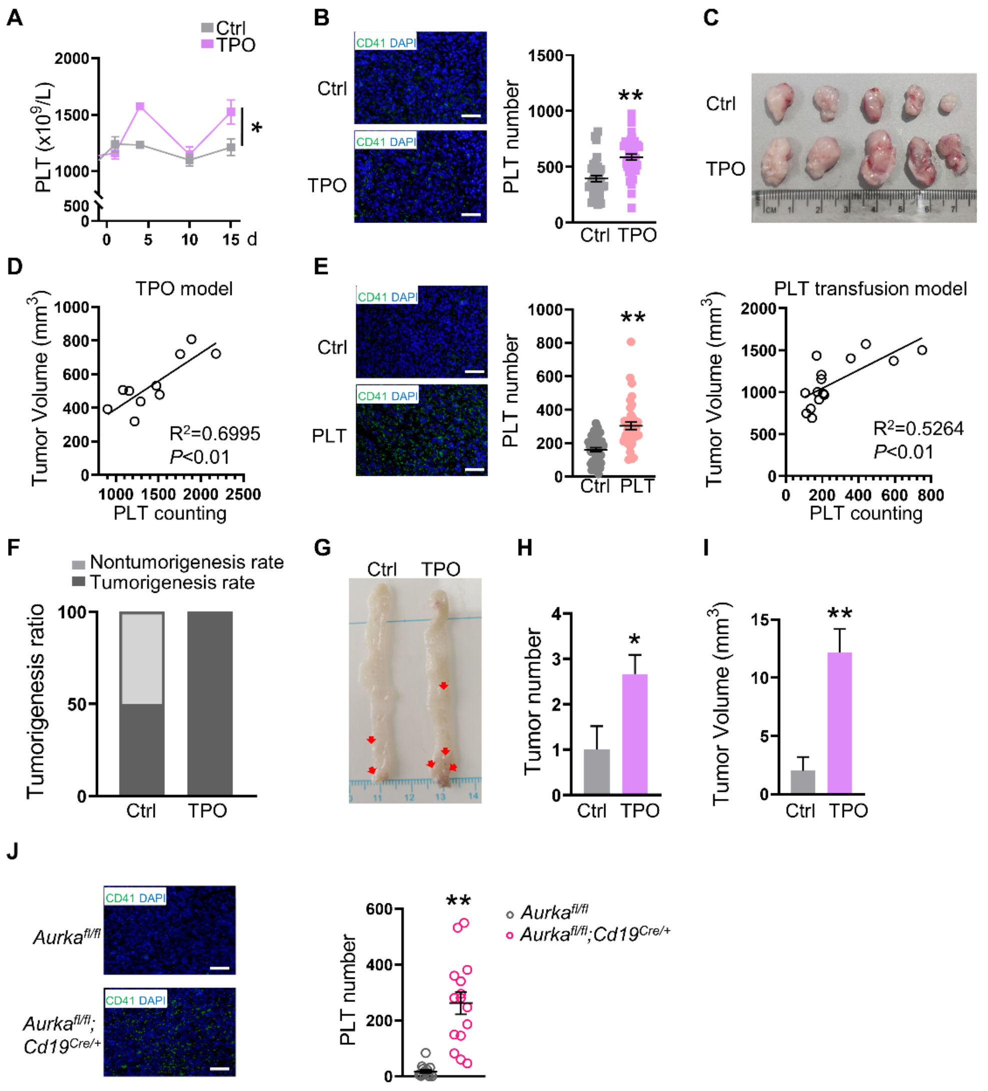

Figure S1 is related to Figure 1. An increase in platelets promoted CRC development.

(A) Mice were treated with or without TPO. Platelet numbers were examined (n = 10). **(B)** Immunofluorescence analysis of CD41 (green) and DAPI (blue) in tumor tissue sections. The number of platelets was counted (right panel). The data are representative of one of two independent experiments (n = 5 mice/group, 8 slides/mouse). **(C)** Image showing tumor volume in a mouse model of TPO-induced platelet elevation (n = 5). (A and C) The data shown are representative of single experiment. **(D)** The number of platelets was positively related to the tumor volume. **(E)** Immunofluorescence analysis of CD41 (green) and DAPI (blue) in tumor tissue sections. The number of platelets was

counted (middle panel) (n = 5 mice/group, 8 slides/mouse). The number of platelets was positively related to the tumor volume (right panel). The data are representative of one of two independent experiments. **(F)** Tumor incidence was assessed. **(G-I)** Image of tumors, the number and size of tumors were assessed in the AOM/DSS-induced CRC model. The data shown are representative of a single experiment. **(J)** Immunofluorescence analysis of CD41 (green) and DAPI (blue) in tumor tissue sections. The number of platelets was counted (right panel) (n = 3 mice/group, 5 slides/mouse). The data are representative of one of two independent experiments. ***P* < 0.01; **P* < 0.05.

Figure S2

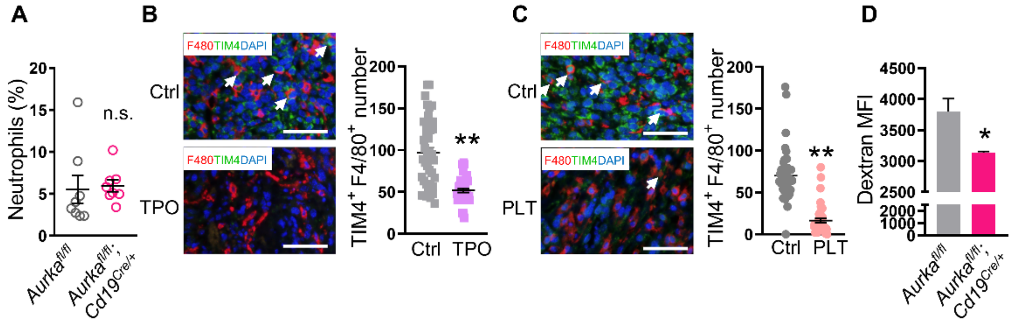

Figure S2 is related to Figure 2. TAMs are the key mediators of platelet-driven tumor development.

(A) The population of Ly6G$^+$ neutrophils was examined by FACS (n = 10). The data shown are representative of a single experiment. **(B-C)** Immunofluorescence analysis of TIM4 (green), F4/80 (red) and DAPI (blue) in tumor tissue sections (n = 5 mice/group, 8 slides/mouse). The number of TIM4-positive TAMs was counted (right panel). The data are representative of a single experiment. **(D)** TAM phagocytosis was assessed by FACS (n = 4). The data are representative of one of two independent experiments. ***P* < 0.01; **P* < 0.05; n.s., not significant.

Figure S3

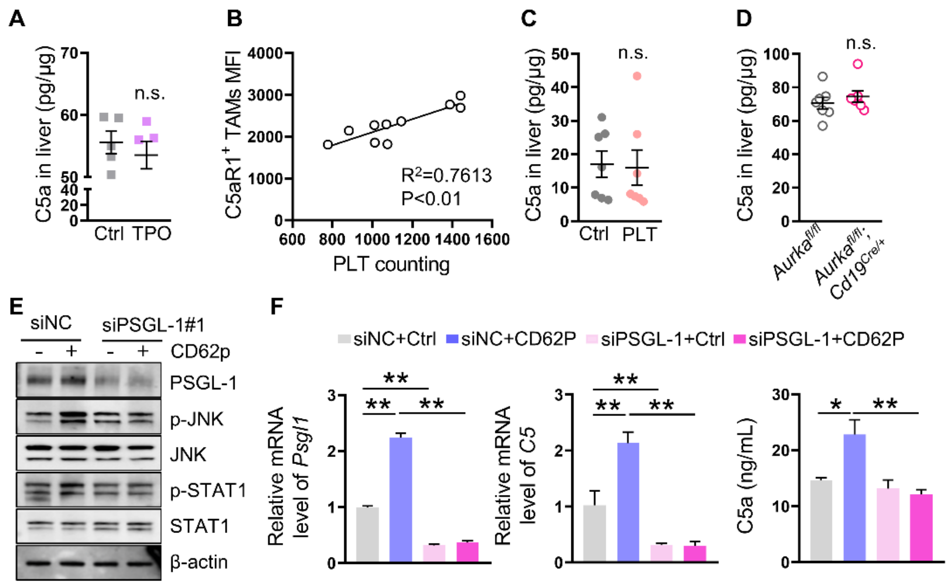

Figure S3 is related to Figures 3 and 4. An increase in platelets promoted TAM C5 transcription via PSGL-1.

(A) The liver level of C5a was measured by ELISA (n = 5). **(B)** The correlation between MFI of C5aR1 on TAMs and the number of platelets was analyzed. **(C-D)** The liver level of C5a was measured by ELISA (n = 7). **(E)** BMDMs were transfected with a negative control siRNA or an siRNA against PSGL-1. After 24 h, cell lysates were harvested and subjected to immunoblotting to examine the indicated proteins. **(F)** Real-time RT-PCR was used to quantify the *Psgl1* and *C5* mRNA levels. The cell culture medium was harvested, and C5a was examined. ***P* < 0.01; **P* < 0.05; n.s., not significant.

Figure S4

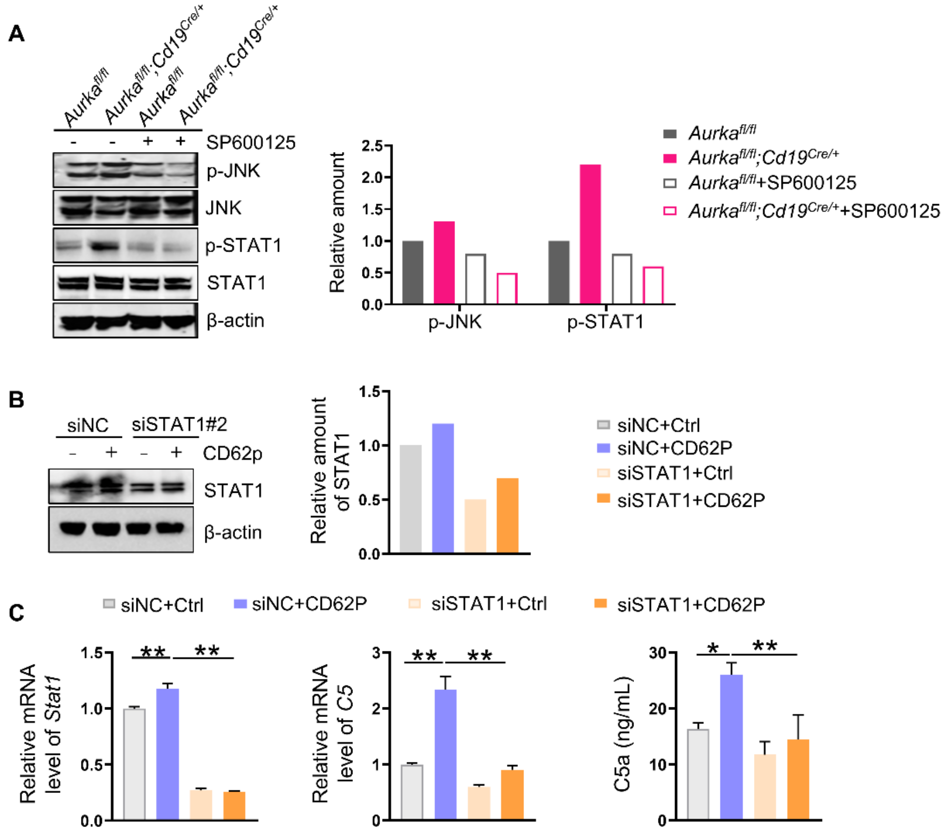

Figure S4 is related to Figure 5. Platelets induce C5 transcription via the PSGL-1 /JNK/STAT1 signaling pathway.

(A) Protein was harvested from *Aurka*$^{fl/f}$ and *Aurka*$^{fl/fl}$;*Cd19*$^{cre/+}$ BMDMs and subjected to immunoblotting. The levels of p-JNK and p-STAT1 were quantified by densitometry, normalized to actin, and plotted (right panel). **(B)** BMDMs were transfected with a negative control siRNA or an siRNA against STAT1. After 24 h, cell lysates were harvested and subjected to immunoblotting to examine the indicated proteins (left panel). The levels of STAT1 were quantified by densitometry, normalized to actin, and plotted (right panel). **(C)** Real-time RT-PCR was used to quantify *Stat1* and *C5* mRNA levels. The cell culture medium was harvested, and C5a was examined (right panel). $^{**}P < 0.01$; $^{*}P < 0.05$.

Figure S5

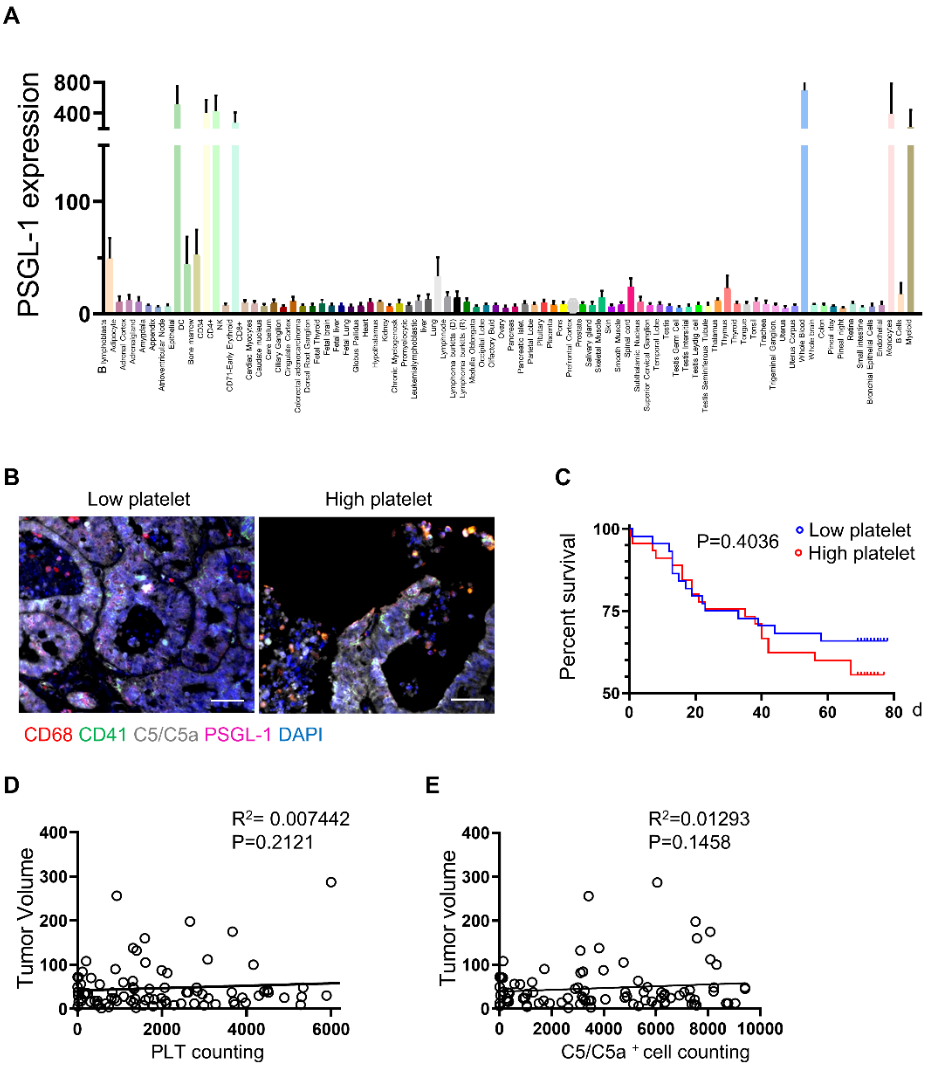

Figure S5 is related to Figure 6. Clinical studies have shown a positive correlation between the number of platelets in the tumor and C5a production.

(A) PSGL-1 mRNA expression in different human cells. **(B)** Immunofluorescence analysis of CD68 (red), CD41 (green), C5/C5a (grey), PSGL-1 (purple) and DAPI (blue)in tumor tissues. **(C)** Survival was analyzed by the log-rank test. **(D)** Correlation analysis of intratumoral platelet counts and tumor size. **(E)** Correlation analysis of the number of intratumoral C5/C5a-positive cells and tumor size.
